# Statins for primary prevention of cardiovascular disease in Germany: benefits and costs

**DOI:** 10.1007/s00392-025-02608-5

**Published:** 2025-03-17

**Authors:** Alexander Dressel, Felix Fath, Bernhard K. Krämer, Gerald Klose, Winfried März

**Affiliations:** 1 D-A-CH-Society for Prevention of Cardiovascular Diseases e. V., Hamburg, Germany; 2 Medical Faculty Mannheim of the University of Heidelberg, Mannheim, Germany; 3 European Center for Angioscience, Medical Faculty Mannheim of the University of Heidelberg, Mannheim, Germany; 4 Drs. T. Beckenbauer and S. Maierhof, Bremen, Germany; 5 Drs. I. van de Loo, K. Spieker and C. Otte, Bremen, Germany; 6 SYNLAB Holding Deutschland GmbH, SYNLAB Academy, Mannheim, Germany; 7 Clinical Institute for Medical and Chemical Laboratory Diagnostics, Medical University of Graz, Graz, Austria; 8 Department of Internal Medicine III (Cardiology, Angiology and Pneumology), Heidelberg University Hospital, Heidelberg, Germany

**Keywords:** LDL cholesterol, Statins, Primary prevention, Risk thresholds, Cost, Effectiveness

## Abstract

**Background:**

The reduction of LDL cholesterol lowers the risk of coronary and cerebrovascular events in individuals without manifest cardiovascular diseases. In Germany, statins at the expense of statutory health insurance had only been permitted for patients with atherosclerosis-related diseases or those at high cardiovascular risk (over 20 percent event probability within the next 10 years, calculated using one of the “available risk calculators”). However, international guidelines recommend lower risk thresholds for the use of statins.

**Methods:**

The health and economic impacts of different risk thresholds for statin use in primary prevention within the German population are estimated for thresholds of 7.5, 10, and 15 percent over 10 years, based on the US *Pooled Cohort Equation* (PCE) which is valid for Germany, using Markov models.

**Findings:**

Cost-effectiveness increases with a rising risk threshold, while individual benefit decreases with age at the start of treatment. The use of statins at a risk of 7.5 percent or more is cost-effective at any age (cost per QALY between 410 and 2100 Euros). In none of the examined scenarios does the proportion of the population qualifying for statin therapy exceed 25 percent.

**Interpretation:**

Lowering the threshold for statin therapy to a risk of 7.5 percent of either non-fatal myocardial infarction, coronary heart disease death, non-fatal or fatal stroke would align statin prescription in Germany with international standards. There is no urgent rationale for applying age-stratified risk thresholds.

**Graphical abstract:**

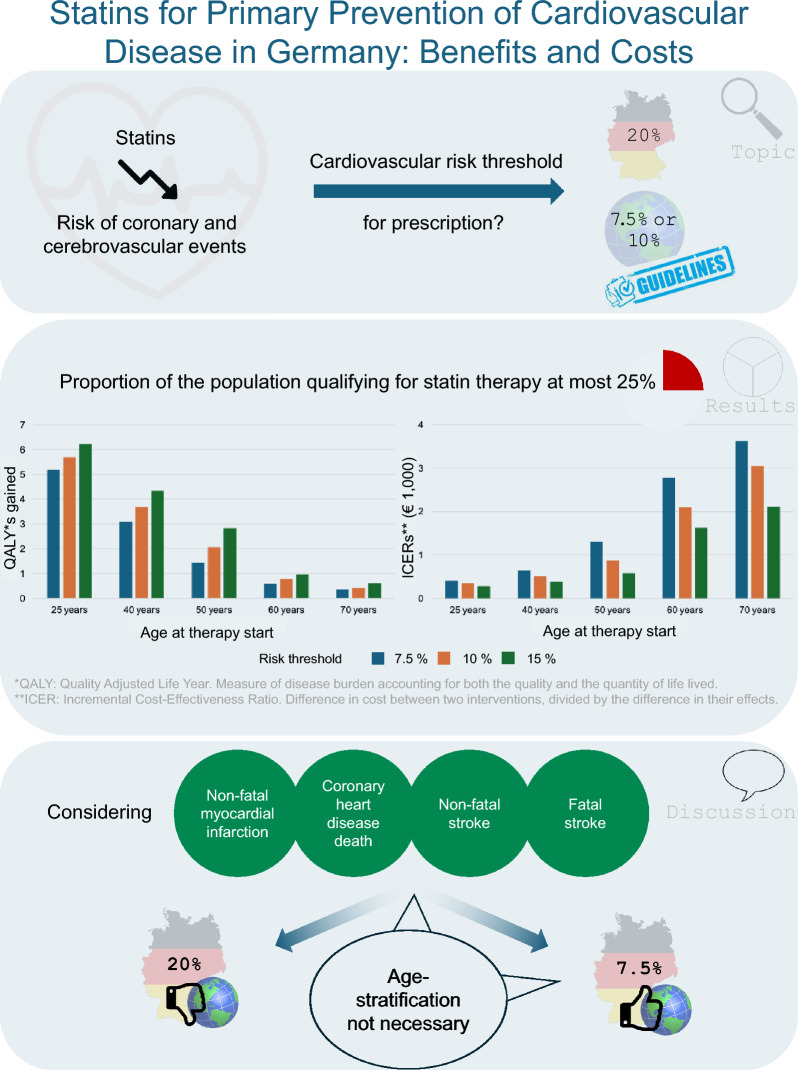

## Introduction

For around two decades, it has been established that lowering **low-density lipoproteins** (LDL) with statins can reduce the incidence of cardiovascular events even in clinically healthy, asymptomatic individuals [1–10].

According to the Cholesterol Treatment Trialists’ (CTT) Collaboration, lowering the concentration of LDL cholesterol (LDL-C) by (absolutely) 1.5 mmol/l (60 mg/dl) will reduce the rate of cardiovascular events by (relatively) around one-third [7]. A recent meta-analysis showed a 25 percent reduction in the rate of serious cardiovascular events and an 11 percent reduction in overall mortality with statins as a class. Expectedly, atorvastatin and rosuvastatin had greater effects [8].

In Germany, it was previously impossible to leverage the potential of statin therapy in cases of moderate cardiovascular risk. This is because, according to Annex III of the Medicines Directive (number 35) of the Joint Federal Committee, lipid-lowering drugs could only be prescribed at the expense of the statutory health insurance in cases of atherosclerosis-related vascular disease (ASCVD) or in “primary prevention” at a cardiovascular risk of over **20 percent event rate in 10 years**, calculated using “one of the available risk calculators” [11]. We pointed out many years ago that the “available risk calculators” lead to widely different assessments of the risk [12]. This finding is consistent with other sources [13] and is partly due to the fact that the predicted clinical endpoints in the risk calculators can be narrow (e.g., cardiovascular death) or broad (e.g., non-fatal myocardial infarction, cardiovascular death, stroke).

Current guidelines consider the risk thresholds for statin use to be well below a 20 percent probability of fatal and non-fatal atherosclerosis-related events in 10 years. The *US Department of Veterans Affairs* and the *US Department of Defense* recommend that patients with a cardiovascular risk of more than 12 percent over 10 years, calculated using the *Pooled Cohort Equation* (PCE [14]), a low-density lipoprotein cholesterol > 190 mg/dl or diabetes mellitus should be offered a moderate-dose statin [15, 16]. The *American College of Cardiology* and the *American Heart Association* suggest treatment with a moderate-dose statin at a risk of 7.5 percent or more [17, 18]. The *National Institute for Health and Care Excellence* (NICE) recommends 20 mg atorvastatin daily at a risk of 10 percent or more in 10 years, calculated using the QRISK3 algorithm [19]. The *European Society of Cardiology* specifies age-dependent risk thresholds. If there is neither diabetes mellitus, chronic kidney disease nor severe dyslipidemia, “very high risk” is assumed for asymptomatic people aged 50 to 69 years if the SCORE2 (Systematic Coronary Risk Estimation 2) [13] is above 10 percent [20]. In the age group under 50 years, a “very high risk” is assumed from 7.5 percent, in the age group over 70 years only from 15 percent, with reference to the high age and competing risks.

As a result of the very similar lowering of intervention thresholds worldwide, the German Federal Joint Committee decided on June 25, 2024 to initiate an opinion procedure to amend Annex III of the Medicines Directive, the subject of which was the review of the outdated risk threshold of 20 percent in 10 years for the prescription of lipid-lowering drugs (www.g-ba.de/AM-RL-III_SN_Nr-35).

In addition, there has been a draft law that aims to enshrine the right to prescribe statins in accordance with the current EAS-ESC guidelines if age-dependent risk thresholds of 7.5, 10 and 15 percent, calculated using the SCORE2 calculator, are exceeded (www.bundesgesundheitsministerium.de/GHG).

The aim of this study is to model the effects of different risk thresholds for the use of statins on the long-term prognosis of the German population, taking health economic aspects into account.

## Methods

We developed Markov models simulating rates of cardiovascular events of 7.5, 10, and 15 percent over 10 years and associated costs and benefits for individuals with no prior history of ASCVD in relation to age at treatment initiation (25, 40, 50, 60, and 70 years) up to age 99 years. The health states included in the models were the occurrence of non-fatal and fatal coronary heart disease, the occurrence of non-fatal and fatal cerebrovascular events as assessed by the PCE [14] and death from other causes; for transitions and their probabilities, see Table 1.

**Table 1:** Model components and assumptions

Component	Acceptance
Markov model	The Markov model is memoryless as the future state depends exclusively on the current state, but not on past states.
State of knowledge	The current state of science and technology is taken into account. Potential future developments are not taken into account.
Study population	Total population of Germany.
Age range	The age of the population included is between 25 and 99 years.
Combination of the sexes	The analysis is carried out on a sex-specific basis. The results for both sexes are presented as a cross-sex average, based on the age-appropriate proportions of the sexes for each year of life [46].
Mortality rate	Mortality rate of the German general population for the years 2017 to 2019 taken from www.destatis.de.
End point	Combined rate of fatal and non-fatal atherosclerosis-related cardiovascular events, calculated from the cause-specific mortality rates and assuming a ratio of non-fatal to fatal coronary events of 2.89 (https://www.helmholtz-muenchen.de/herzschlag-info) and of non-fatal to fatal cerebrovascular events of 3.31 [21].

Event types and probabilities	The following events are taken into account: a) non-fatal coronary events, b) non-fatal cerebrovascular events, c) fatal coronary events, d) fatal cerebrovascular events, e) fatal events that are neither coronary nor cerebrovascular. A person with a history of ASCVD is a person who is alive and has suffered at least one non-fatal coronary event or at least one non-fatal cerebrovascular event. Otherwise, it is a person who is alive but has no previous history of ASCVD. The possible states are: i) (alive) without history of ASCVD, ii) (alive) with history of ASCVD, iii) dead, whereby events a) and b) transform state i) into state ii) while leaving state ii) unchanged. Events c), d), and e) lead from state i) as well as from state ii) to state iii). For individuals with a history of ASCVD, the probability of a coronary (or cerebrovascular) event is c1 times (or c2 times) that of an individual without a history, whereby c1 = 1.444 and c2 = 2.666 [47], respectively. The presence of a history thus modifies the probability of a subsequent event once, but not repeatedly.
Number of non-fatal events	When classifying the risk of a fatal coronary or cerebrovascular event, only the existence or absence of a history of previous events is taken into account. The number of non-fatal CHD or CVE events in the history of previous events is not taken into account.
Combination of risk types	Transitions between different event types are not taken into account. This means that patients with a non-fatal CHD event cannot suffer a non-fatal or fatal CVE event in future and vice versa.
Costs due to events	Only direct event costs rounded to the nearest one are taken into account: - fatal CHD event, 10,671 Euros, - non-fatal CHD event, 20,215 Euros, - fatal cerebrovascular event, 11,874 Euros, - non-fatal cerebrovascular event, 24,044 Euros. The calculation of event costs is based on the published data [26].
Risk stratification	Risks for the combined endpoint of 7.5, 10 and 15 percent in 10 years, calculated according to the PCE [14].

Intervention	The rate of fatal and non-fatal coronary and cerebrovascular events is reduced by 30 percent with statin treatment in every risk and age stratum, regardless of sex [22, 23].
Cost of statins	Direct and rounded annual costs for statin therapy without possible discounts of 100 Euros per year are applied. This corresponds approximately to the cost of 40 mg rosuvastatin daily (LAUER-TAXE®). The price is dated 15.10.2023.
Utility weights	- 1.0 with no coronary or cerebrovascular event, - 0.9648 (0.9505–0.9758) after at least one coronary event and without cerebrovascular events, - 0.8835 (0.8414–0.9108) after at least one cerebrovascular event and without coronary events, - 0.8524 (0.7998–0.8888) after at least one coronary and at least one cerebrovascular event (with confidence intervals in parentheses) [24, 25].
Adjusting the quality of life	The use of drug therapy has no effect on the quality of life.
QALYs (quality-adjusted life years)	Years of life × benefit weights.
ICER (incremental cost-effectiveness ratio)	Costs of statin treatment / QALYs gained.
Start of treatment	Treatment starting points are assumed at the ages of 25, 40, 50, 60 and 70 years.
Adherence	Treated patients are adherent.
Discounting	The annual discounting of 3 percent is taken into account.

**Fig. 1:**
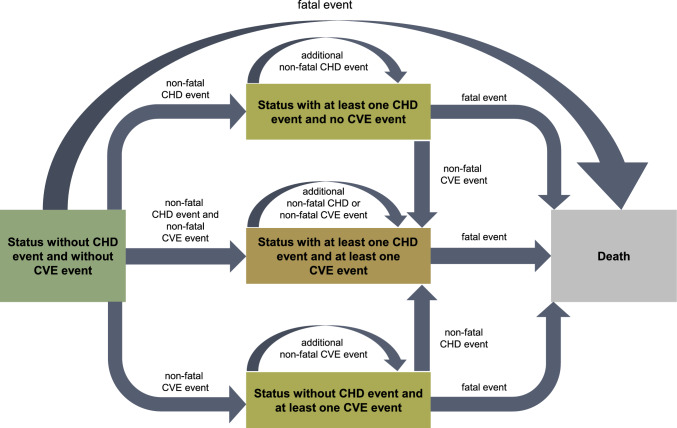
Cohort state transition model for clinically asymptomatic individuals. The occurrence of events was calculated using the Markov chain Monte Carlo method with a cycle length of one year and 20,000 bootstrap iterations up to a maximum age of 99 years. The transition probabilities were estimated from the mortality rates stratified by age cohort and cause of death using fixed ratios of non-fatal to fatal coronary and cerebrovascular events and adjusted to the survival data of the German general population available at www.destatis.com. In addition, the theoretical effects of lipid-lowering treatment with statins were taken into account. Non-fatal coronary and cerebrovascular events may coincide within any one-year period. Only one non-fatal coronary or cerebrovascular event is included in the calculation of the additional risk of patients with a history of coronary or cerebrovascular events; whether such an event occurs once or more frequently has no influence on the additional risk

The calculation of the event probabilities is based on the mortality rates of the general population in Germany. To exclude special effects caused by the Corona virus pandemic, we selected data from the period 2017 to 2019 (www.statistischebibliothek.de). Fatal and non-fatal events were calculated by combining this basis with non-fatal to fatal ratios. For example, we used the ratios 2.89 (www.helmholtz-muenchen.de/herzschlag-info) and 3.31 [21] for obtaining non-fatal coronary and cerebrovascular events, respectively. Event rates were simulated using Markov chain Monte Carlo (MCMC) methods with a cycle length of one year and 20,000 bootstrap iterations up to a maximum age of 99 years (Fig. 1).

For different scenarios, we estimated the numbers of people needed to treat to prevent an event (NNT), based on a treatment period of 5 years (the most common approximate duration of statin outcome trials), the quality-adjusted life years (QALYs) gained per person, savings from the avoidance of coronary and cerebrovascular events, incremental cost-effectiveness ratios (ICERs), the total costs of implementing these scenarios (budget impact) and their effects on mortality in the German population. We assumed a relative risk reduction of 30 percent through treatment with statins [22, 23]. A glossary of the most important measures in health economy is provided with Table 2.

**Table 2:** Glossary of technical terms

Term	Explanation
Cost-effectiveness	Cost-effectiveness is an economic evaluation method that compares the costs and health outcomes of different interventions or treatments to determine which provides the best results for the resources invested. In healthcare, cost-effectiveness analysis (CEA) helps decision-makers understand whether a new treatment or intervention offers good value for its cost relative to existing options.
Discount rate	Costs and health outcomes occurring in future are usually valued less than at present. This is accounted for by discounting costs and savings. The use of a higher discount rate results in less value being attached to costs and health outcomes in future.
Incremental cost-effectiveness ratio (ICER)	Difference costs between two interventions, divided by the difference in their effect. It indicates the average incremental cost per unit of effect, which is here one Quality-Adjusted Life Year (QALY) gained.
Markov chain Monte Carlo	Markov chain Monte Carlo (MCMC) is a method to sample from complex probability distributions by creating a sequence (chain) of samples. This chain gradually approximates the target distribution, allowing us to estimate its properties, even when direct sampling isn’t feasible.
Markov model	A Markov model is a mathematical model used to analyze systems that transition between various states over time, especially useful in health economics and decision analysis. It is particularly suited for scenarios in which an individual or system can move through different health states (such as healthy, sick, or deceased) with certain probabilities and in which future states depend only on the current state (i.e., the Markov property).
Numbers Needed to Treat (NNT)	Number Needed to Treat (NNT) is a statistical measure used in healthcare to assess the effectiveness of a treatment or intervention. Specifically, NNT represents the average number of patients who need to be treated to prevent one additional adverse outcome (such as a stroke, heart attack, or death) or to achieve one additional positive outcome.
Quality-Adjusted Life Years (QALY)	Measure of disease burden accounting for both the quality and the quantity of life lived. One QALY corresponds to one year in perfect health. If an individual’s health is compromised by a chronic disease state QALYs are accrued at a rate of less than 1 per year.
Utility weight	Factor to adjust QALYs for the state of health. A year of life in perfect health is worth 1.0. A year of life while suffering from a chronic disease is worth less.

The utility weights were derived from the literature (Table 1) [24, 25]. The estimation of direct medical costs is based on published data [26]. The cost of statin therapy was conservatively assumed to be 100 Euros per year. Both costs and benefits were discounted at an annual rate of 3 percent to discount future to today’s value. Indirect costs and benefits were not taken into account (Table 1).

All analyses were conducted separately for men and women; costs, QALYs, and life years gained were calculated as cross-sex averages in order to derive the ICERs in particular. The proportions of men and women in the German population in 2022, stratified by age cohort, were used as a basis (www.destatis.com).

The Markov model was implemented using the software R (version 4.2.2).

## Results

**Fig. 2:**
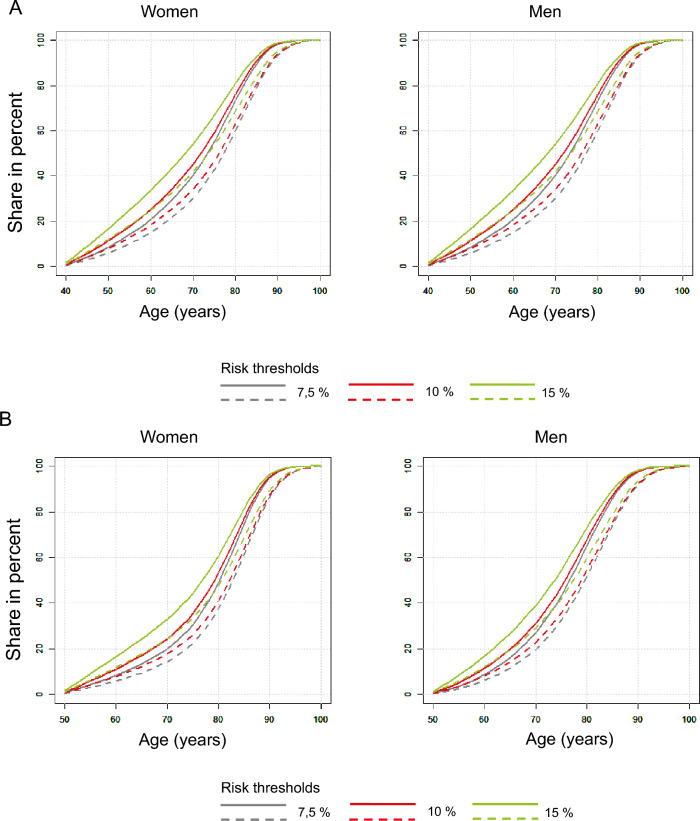

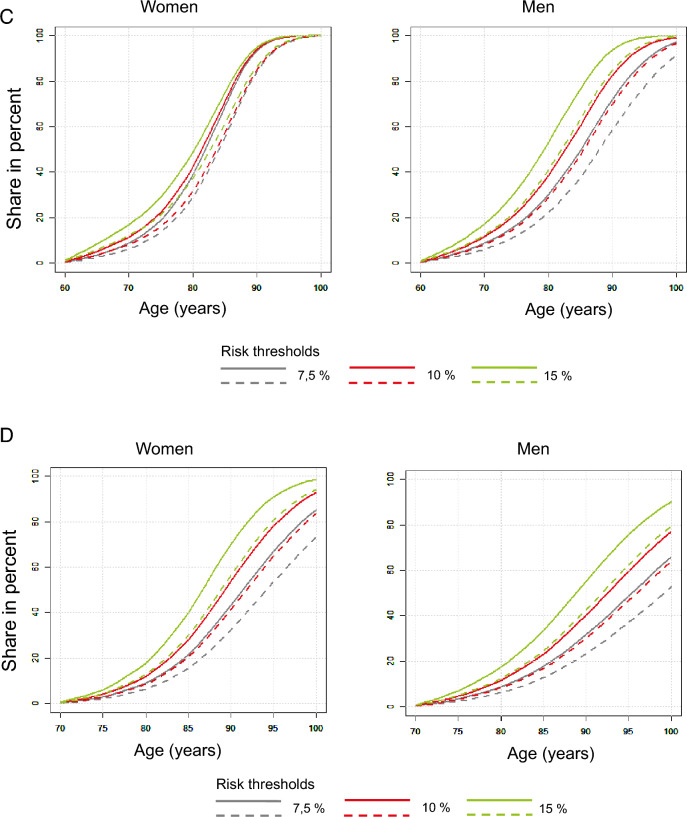
Estimated percentage of persons among the living with coronary or cerebrovascular events over time. Inclusion condition: no history of coronary or cerebrovascular events. The *solid* lines show the percentage of untreated living individuals with at least one coronary or cerebrovascular event in the population strata, the *dashed* lines show the percentage of statin-treated and living individuals. 10-year risks at the start of treatment 7.5 (*blue*), 10 (*red*) and 15 (*green*) percent. Start of treatment at the earliest age of 40 (*2A*), 50 (*2B*), 60 (*2C*) or 70 (*2D*) years. *Left:* women; *right:* men

The survival curves in Fig. 2 (A to D) show the percentage of people among the living who suffer at least one coronary or cerebrovascular event over time for the scenarios of starting therapy at the age of 40, 50, 60 and 70 years and the 10-year risks of 7.5, 10 and 15 percent if there was no history of coronary or cerebrovascular events before starting therapy. As expected, the proportion of patients suffering an event increases with age. The figure also illustrates the effects of lipid-lowering intervention with statins on the risks in the age strata.

The *numbers needed to treat* (NNTs) in Table 3 are based on 5 years rather than 10 years as this corresponds approximately to the duration of most prospective, randomized and placebo-controlled studies with statins. The NNTs decrease in parallel to the risk threshold. In contrast, they increase when treatment is started later in life. The lowest NNT (24 in 5 years) is achieved with a risk threshold of 15 percent and treatment starting at the age of 25, the highest one with a risk threshold of 7.5 percent, and the treatment starting at the age of 70 (NNT 429).

**Table 3:** Effects and costs of reducing the risk of fatal and non-fatal coronary and cerebrovascular events by 30 percent in Germany

Risk threshold (percent per decade)	Age at start of therapy (years)	NNT (5 years)	QALYs gained with 95% CI (years)	ICERs (1000 Euros)	Avoided event costs discounted (1000 Euros)	Share of total population (%)	Mean risk of the treated population	Annual budget (Euro billion)	Increase in life expectancy, treated population (years)	Increase in life expectancy, total population (years)
7.5	25	48	5.19 (4.80; 5.57)	0.41	1.51	23.20	11.72	1.93	-*	-*
7.5	40	58	3.07 (2.78; 3.36)	0.64	1.87	22.98	17.57	1.91	1.29	0.30
7.5	50	194	1.43 (1.21; 1.65)	1.31	2.14	21.97	16.22	1.83	1.17	0.26
7.5	60	372	0.59 (0.39; 0.78)	2.78	2.50	16.36	18.00	1.36	0.79	0.13
7.5	70	429	0.36 (0.20; 0.52)	3.62	1.38	7.10	29.30	0.59	0.34	0.02
10	25	36	5.68 (5.30; 6.06)	0.35	1.55	19.16	13.53	1.59	-*	-*
10	40	41	3.68 (3.39; 3.98)	0.51	1.96	19.02	22.56	1.58	1.41	0.27
10	50	77	2.06 (1.83; 2.29)	0.87	2.12	18.41	21.82	1.53	1.32	0.24
10	60	276	0.77 (0.58; 0.95)	2.10	3.00	14.59	21.23	1.21	0.96	0.14
10	70	318	0.42 (0.26; 0.58)	3.05	1.65	6.71	30.60	0.56	0.43	0.03
15	25	24	6.22 (5.86; 6.58)	0.28	1.59	13.85	16.60	1.15	-*	-*
15	40	26	4.33 (4.04; 4.62)	0.38	2.13	13.77	29.31	1.15	1.56	0.21
15	50	36	2.82 (2.58; 3.06)	0.58	2.07	13.50	30.08	1.12	1.51	0.20
15	60	219	0.96 (0.78; 1.14)	1.63	2.79	11.50	28.68	0.96	1.25	0.14
15	70	208	0.61 (0.45; 0.76)	2.11	2.25	5.94	34.20	0.49	0.62	0.04

^*^The database is not sufficient to calculate these values

*Risk of 7.5 percent per 10 years.* For people with a risk of 7.5 percent or more in 10 years at the age of 25, a lifelong risk reduction of 30 percent (with a statin) leads to a gain of 5.19 QALYs; 410 Euros must be spent for one QALY. In this scenario, around 23 percent of the population aged 25 years or older has a risk of more than 7.5 percent; 77 percent of the population are not treated (Table 3). The proportion of the total population receiving treatment decreases substantially only if the start of treatment is shifted to the sixtieth year of life or older as only a few young people exceed the risk threshold of 7.5 percent. The proportion of people receiving treatment only falls below 10 percent of the population when treatment is started from the age of 70 (Table 3).

*Risk of 10 percent per 10 years.* The cost-effectiveness increases as expected if the risk threshold is raised to 10 percent. A 25-year-old treated person then gains slightly more QALYs (5.68) compared to the same person with a risk of 7.5 percent (5.19). The ICER is reduced from 410 to 350 Euros. The proportion of people in need of treatment (at 10 percent or more risk and 25 years and older) in the total population is 19 percent. This decreases significantly only from the age of 60 onwards.

*Risk of 15 percent per 10 years.* There is an even greater individual benefit for people with a risk of 15 percent and above: If risk reduction is started at the age of 25, a gain of 6.22 QALYs can be expected at a cost per QALY of only 280 Euros. Around 14 percent of the population will be treated and 86 percent will be excluded from treatment.

**Fig. 3:**
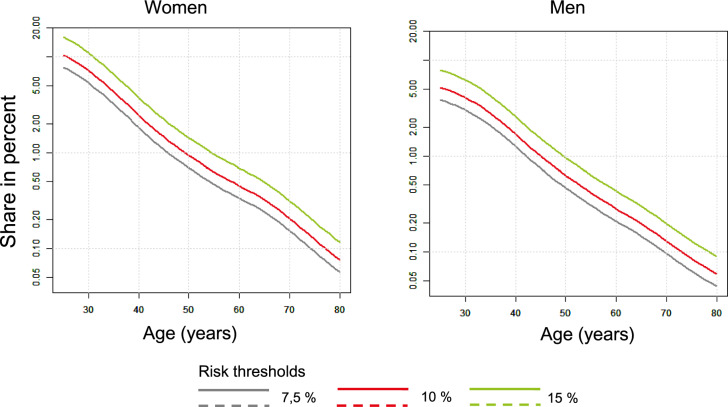
Influence of age on the ratios of the 10-year risk of fatal coronary or cerebrovascular events to the 10-year risk of death from other causes. The ordinate is logarithmically scaled. The lines show the progression of the ratios in the population strata with 10-year risks from 7.5 (*blue*), 10 (*red*) and 15 (*green*) percent for cardiovascular events calculated according to the *Pooled Cohort Equation* (PCE). *Left:* women; *right:* men

Remarkably, the absolute benefit of the risk reduction diminishes in parallel with the age at the start of treatment. At the age of 70, with a risk threshold of 7.5 percent, a gain of only 0.36 QALYs can be expected at a cost of 3620 Euros per QALY. With risk thresholds of 10 and 15 percent, the gains are 0.42 and 0.61 QALYs, and incremental costs of Euros 3050 and Euros 2110 are incurred. The diminishing benefit of treatment arises from *competing risks*, the magnitude of which we have shown as the ratio of cardiovascular deaths to deaths from non-cardiovascular causes in Fig. 3. For women aged 30, this ratio is around 50 and falls to 0.5 by the age of 80. For men aged 30, it is around 30 and also decreases to around 0.5 with age.

### Budget impact

The annual costs from the perspective of the payers depend on the choice of risk threshold and the time of treatment start. They range between Euros 0.49 billion (risk threshold of 15 percent and start of treatment from the age of 70) and Euros 1.93 billion (risk threshold of 7.5 percent and start of treatment from the age of 25). For all risk thresholds, treatment costs only fall significantly when treatment begins at the age of 60 or later.

The treatment costs are offset by an increase in average life expectancy both in the treated group of people and in the population as a whole. Assuming treatment to begin at the age of 40 and a risk threshold of 7.5%, this amounts to 1.29 years in the treated population (23% of the total population) and 0.30 years in the total German population (treated and untreated). The increase in the average life expectancy of a treated population consisting of people above a certain age is lower than the gain in QALYs of an individual who starts treatment at a certain age. This is because starting treatment at a certain age implies that all older people with higher risks are also treated. This is illustrated by looking at the mean risk of the population to be treated (Table 3, column 9).

## Discussion

We have modeled the potential effects of primary prevention with statins based on the available evidence from randomized, placebo-controlled intervention trials and against the background of the current German healthcare landscape. In Germany, costs per QALY of less than 40,000 Euros (roughly equivalent to gross domestic product per capita) are considered “very cost-effective” [27], costs per QALY of up to 120,000 Euros are still considered cost-effective. In all scenarios examined, the prescription of statins in primary prevention would be highly cost-effective from a risk threshold up to the “most unfavorable” constellation, namely statin therapy from a risk threshold of 7.5 percent in 10 years from the age of 70. There is therefore no compelling justification, at least for statin therapy, to make the risk threshold dependent on age, as proposed by the ESC without explicit justification [20].

*Competing risks.* Based on our results, the competing risks increase almost 100-fold compared to an age of 30 years. The concept of competing risks implies that the risk of a particular event, in this case death from cardiovascular causes, is reduced by the increase in other competing causes of death. These considerations would potentially provide an unambiguous rationale for the ESC’s recommendation to raise the risk threshold for initiating statin therapy in parallel with age. Such a recommendation would concededly be justified for significantly more costly treatment approaches than statins. However, since starting statin therapy in older people at the risk threshold of 7.5 percent can still be classified as “very cost-effective”, we advocate accepting the low risk threshold of 7.5 percent in this group as well. This is also because stratification by age would probably create unnecessary confusion rather than clarity in practice.

*Size of risk reduction.* Our findings are based on the assumption that the cardiovascular risk is reduced by 30 percent through statin therapy. According to the Cholestrol Treatment Trialists (CTT) Collaboratiom [7], an absolute reduction of LDL-C by 1 mmol/l translates into a relative risk reduction by approximately 20 percent. A 30 percent risk reduction then requires a long-term lowering of LDL-C by 1.5 mmol/l (60 mg/dl). At an average LDL-C of around 3.34 mmol/l (129 mg/dl) in the German population [28], a relative reduction of LDL-C of 45 percent would be needed which this is ambitious, but achievable with intensive statin monotherapy [29] although it would of course require long-term adherence. Sensitivity analysis not presented here already shows that even less optimistic assumptions hardly compromise the cost-effectiveness of statin therapy.

*Cost-effectiveness of statins in perspective.* The cost-effectiveness of low-threshold and early statin therapy is significantly better than that of other widely accepted non-medical life-saving measures. For example, the average cost of a QALY saved by preventing workplace exposure to pollutants is reported to be around USD 1.4 million, while the cost of containing pollutants in the environment is reported to be USD 4.2 million [30].

The widespread implementation of statin therapy in Germany has repeatedly been opposed by fears of the “medicalization” of the population, and references to non-drug interventions such as exercise or a "heart-healthy" diet have been made. Their potential has been known for many years and certainly remains undisputed. However, the sustainable implementation of lifestyle modifications, for whatever reason, has had little success to date. Germany is one of the countries with the highest LDL-C concentrations in the world [31]; the prevalence rates of overweight, obesity and diabetes mellitus are increasing, especially compared to the turn of the millennium [32, 33].

Finally, it should not be disregarded that lifestyle changes can also be costly. A cost of USD 1915 per QALY was reported for a guideline-based, structured smoking cessation program [35]. The cost of preventing diabetes mellitus through intensive lifestyle modification is likely to be between USD 6000 and USD 13,000 per QALY [36–38], but has also been reported in the order of USD 50,000 per QALY [39]. Regardless of costs, it is ultimately questionable whether the resources required for the implementation and follow-up of sustainable life-style measures would even be available in the German healthcare system. According to strict health economic criteria, lifestyle interventions are therefore not necessarily preferable to the low-threshold prescription of statins.

*Sensitivity to the choice of the risk calculator.* Clearly, the proportion of people who qualify for statin prescribing depends on which calculator is used to determine risk. We have previously observed considerable differences between the commonly used risk algorithms, which are partly due to differences in the predicted endpoints. For example, the one used here and validated for Germany [12, 40] provides the probability of the first occurrence of non-fatal myocardial infarction, fatal coronary heart disease or any stroke. The ESC-SCORE used from 2003 onwards (hereinafter referred to as SCORE1 [41]), provides the probability of cardiovascular death only. Therefore, depending on the study, the SCORE1 was usually less than half as high as the risk according to the PCE. [12, 13] Progress could be achieved through international harmonization of the most important risk calculators [13]. In the meantime, SCORE1 (cardiovascular death) has been followed by SCORE2 [42], the endpoint of which has been set equal to the combined endpoint of the PCE by "multiplicative" adjustment (page 3250 in reference [20]). Some uncertainty still remains as to whether SCORE2 really improves risk prediction over SCORE1 [43]. This uncertainty, however, only limits our estimates of the proportion of the population receiving treatment rather than other essential results.

*Proportion of patients eligible for treatment.* The proportion of people qualifying for statin treatment do not exceed 25 percent of the total population in any of our scenarios. This corresponds to the proportions of 22 to 25 percent reported by Pennells et al. [13] after recalibration of the risk calculators. They are even lower than the figures reported by Mortensen [43] based on the PCE with a risk threshold of 7.5 percent in the Danish population and in the order of 26 percent according to the recommendations of UK-NICE [43].

To the contrary, the application of the age-dependent risk thresholds of the SCORE2 would imply that only 4 percent of the population and only 1 percent of women would qualify for treatment. According to Mortensen et al. [43], lowering the risk threshold of the SCORE2 to 5 percent would be roughly equivalent to a risk threshold of 7.5 percent according to PCE. These considerations suggest that further research into the most reliable risk equation for the German population which is outside the scope of the current work is needed.

*Public health implications.* Germany is the European leader in regard to the economic burden of cardiovascular diseases, whereas the average spending is 630 Euros per capita across Europe, and the burden in Germany is at 903 Euros [34]. In relation to the overall costs of healthcare, life expectancy in Germany is disappointingly low, which has been attributed to deficits in cardiovascular prevention [44]. We here show that low threshold and early treatment with statins at least have the potential to increase the average life expectancy of the population. This of course applies above all to the population treated with statins while the average life expectancy of the population as a whole is increased to a lesser extent. In any case would our current results justify a broad implementation of statin treatment which was among the objectives of the draft for the “Healthy Heart Act” that has not been passed at the time of this writing. Regardless of whether this law will come into force, our work has clear messages:

- In its current state, the German healthcare system is not prepared to respond adequately to the challenges of cardiovascular diseases.
- Fundamental and significant changes must occur in Germany to successfully cope with cardiovascular diseases.
- Lower thresholds for the use of statins early in life are warranted.
- Such thresholds can be defined by the German Federal Joint Committee regardless of legal regulations.
- Although we focus on the German healthcare system here, our results may apply to other countries, at least industrial ones. For instance, risk thresholds can be used universally if country- and region-specific risk algorithms are used and sensitivity analysis for pricing and costs of avoided events is easily conducted.

Overall, the annual costs associated with the proposed strategy range between 0.49 and 1.93 billion Euros (Table 3, column 8) and thus between 0.5 and 4 percent of the current expenditure for drugs (around 50 billion Euros) [45].

The authors can only mention here the dilemma that, given limited resources for healthcare, other, previously reimbursable services which are obviously less effective *quoad vitam* may have to be restricted in future. Such a prioritization of healthcare services based on key health economic data will also depend on the results of a transparent social discourse that is free from vested interests and is beyond the scope of this article.

We conclude that the low-threshold prescription of moderately to highly effective statins is a cost-effective option for the primary prevention of cardiovascular disease. Although age-dependent risk thresholds are basically plausible, for pragmatic reasons, we strongly recommend  a risk threshold of 7.5 percent in 10 years across all age strata which is in line with US recommendations.

Finally, we emphasize that our work is subject to the limitations of any health economic analysis. It is a simulation based on assumptions that do not take into account uncertain factors, such as medical progress (e.g., gene therapy), disease progression, cost trends or patient preferences. The results are therefore merely a schematic decision-making aid for medical professionals and other stakeholders in the healthcare system. The benefits and the risks of statin therapy must be weighed by the practitioner in each individual case even if the long-term side effect potential of statins is low compared to the clinical benefit [7].

Finally, it does not seem opportune to us to enshrine the right to statin treatment in law and ignore other important pillars of primary prevention, such as weight reduction, treatment of diabetes mellitus and hypertension. Statin therapy is undoubtedly effective and cost-effective at a lower than the previous global risk threshold of 20 percent in 10 years.. The open question is whether society wants to afford such a therapy. What is certain, however, is that "Western" healthcare systems currently afford much more expensive therapeutic measures without serious concerns about “cost-effectiveness”.

## Data Availability

Data, references, and referrals not provided through the manuscript may be based on secondary use of health data. Such data may be made available to researchers upon request and approval. This procedure makes sure that rules of good scientific practice are followed, and that credit is given to the people who have been in charge of the design and the organization of the study. Interested researchers are invited to address their request or proposal to Felix Fath (felix.fath@carehigh.de) or to the senior author of this, Winfried März (winfried.maerz@synlab.com).
